# The impact of aspirin on *Klebsiella pneumoniae* liver abscess in diabetic patients

**DOI:** 10.1038/s41598-020-78442-8

**Published:** 2020-12-07

**Authors:** Chien-Hsiang Tai, Chien-Ning Hsu, Shih-Cheng Yang, Cheng-Kun Wu, Chih-Ming Liang, Wei-Chen Tai, Seng-Kee Chuah, Chen-Hsiang Lee

**Affiliations:** 1 Division of Infectious Diseases, Department of Internal Medicine, Kaohsiung Chang Gung Memorial Hospital, Kaohsiung, Taiwan; 2 Department of Pharmacy, Kaohsiung Chang Gung Memorial Hospital, Kaohsiung, Taiwan; 3 School of Pharmacy, Kaohsiung Medical University, Kaohsiung, Taiwan; 4 Division of Hepato-Gastroenterology, Department of Internal Medicine, Kaohsiung Chang Gung Memorial Hospital, Kaohsiung, Taiwan; 5 College of Medicine, Chang Gung University, Kaohsiung, Taiwan

**Keywords:** Clinical microbiology, Bacterial infection

## Abstract

In this study, we aimed to investigate the impact of aspirin on the risk of pyogenic liver abscess caused by *Klebsiella pneumoniae* (KP-PLA) and invasive KP-PLA syndrome (IKPS) in diabetic patients. Diabetic patients who were propensity-score matched were retrospectively included from hospital-based database. Kaplan–Meier approach with a log-rank test was used to compare the cumulative incidences of KP-PLA including IKPS between aspirin users and non-users. Totally, 63,500 patients were analyzed after propensity-score matching (1:1). Compared with that of non-users, the incidence of KP-PLA was significantly reduced in aspirin users (0.31% vs. 0.50%, *p* < 0.01), but not for that of IKPS (0.02% vs. 0.03%, *p* = 0.29). Patients taking aspirin for ≥ 90 days had a significantly lower risk for KP-PLA (hazard ratio, 0.67; 95%CI, 0.50–0.90). Females, taking clopidogrel or metformin for ≥ 90 days, and taking H2-blockers or proton pump inhibitors (PPIs) for ≥ 5 days were also associated with a lower risk of KP-PLA. However, cholangitis and a glycated hemoglobin ≥ 8.5% were associated with an increased risk of KP-PLA.

## Introduction

Pyogenic liver abscess (PLA), an infection of the liver parenchyma, is a potentially life-threatening disease, the incidence of which is increasing in many countries such as the US, Denmark and Taiwan^1–3^. Diabetes mellitus (DM), liver cirrhosis, and advanced age are known risk factors for PLA^4–6^. Among these at-risk individuals, diabetic patients tend to have more serious complications of PLA, for which *Klebsiella pneumoniae* (KP) has been shown to be the leading pathogen^7,8^. The majority of KP isolates that cause PLA (KP-PLA) are of K1 and K2 capsule serotypes, which account for 60–70% and 5–20% of the cases reported in Southeast Asia, respectively^9–11^. Invasive KP-PLA syndrome, including PLA and metastatic infections involving unusual distant sites such as the eye, brain, lung, or prostate, have also been observed in diabetic patients, especially in those with poor glycemic control^12^.

Nuclear factor κB signaling and the interleukin-12/interferon-gamma signaling pathway are disrupted in diabetic animals and patients, which might play an important regulatory role in the development of KP-PLA^13,14^. Furthermore, *in-vitro* studies revealed that, in high glucose media, KP serotypes K1 increased gene expression and increased synthesis of the capsular polysaccharide (CPS), which is an important virulence factor for KP^15,16^. It was also observed that phagocytosis of KP serotypes K1 and K2 by neutrophils was impaired in type 2 diabetic patients with poor glycemic control, and this was associated with an increased risk of metastatic complications^16,17^. An unsubstantiated but biologically plausible mechanism for this observation is that metastatic seeding by KP with specific serotypes during bacteremia is due to DM-related loss of vascular patency or integrity.

Therapeutic drugs administered for some chronic diseases may affect the recurrence of PLA in diabetic patients. Our previous study have demonstrated that aspirin could reduce the recurrence rate of PLA^18^. Aspirin can trigger lipoxin and enhances macrophage phagocytosis of bacteria^19^. We previously also reported that aspirin enhanced the susceptibility of KP serotype K1 to leukocyte phagocytosis by reducing CPS production; moreover, our multivariate analysis revealed that patients with community-acquired KP bacteremia who had recently used aspirin were at a lower risk of acquiring invasive KP-PLA syndrome^20^. Beyond the general understanding that DM is a risk factor for the development of invasive KP-PLA syndrome, little is known about how aspirin affects its development in diabetic patients. To the best of our knowledge, no large-scale population-based study has previously been performed to investigate the association between KP-PLA, including invasive KP-PLA syndrome, and aspirin use.

The primary aim of the current study was to determine the role of aspirin use in diabetic patients and its association with the risk of KP-PLA and invasive KP-PLA syndrome.

## Results

### Clinical characteristics of diabetic patients with and without aspirin use

**Figure 1:**
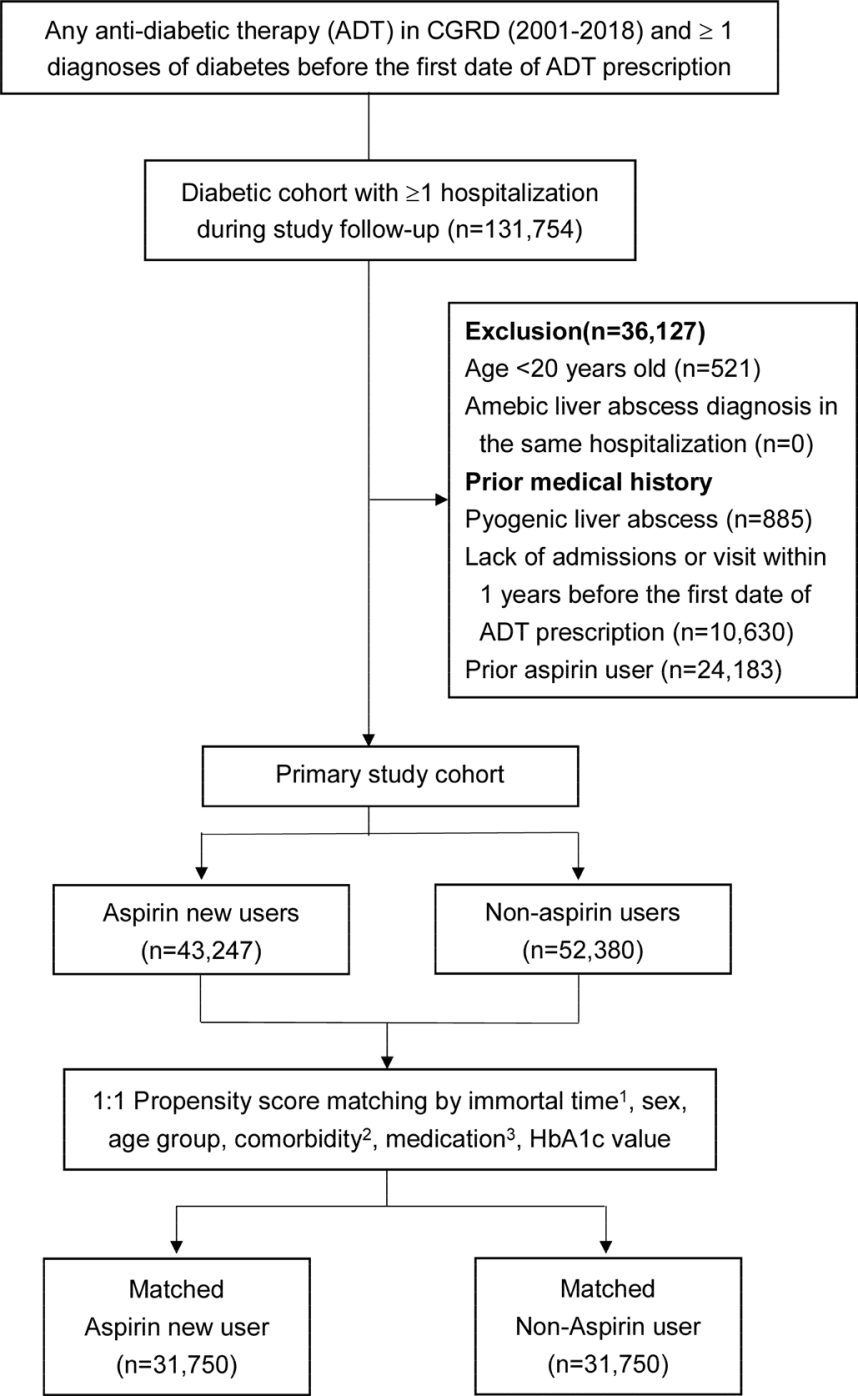
Flow chart of patient selection. 1. Immortal time: Patients who received aspirin were propensity matched in a 1:1 ratio with those who were not taking aspirin and were alive at the time of the first dispensed prescription to their matched counterpart. 2. Comorbidities: cirrhosis, injury to the liver, hepatobiliary malignancy, gastric cancer, colon cancer, cholangitis, bile duct stones/cholelithiasis, viral hepatitis, hepatitis B, hepatitis C, renal disease, hepatocellular carcinoma, metastatic cancers, other malignancies, ischemic heart disease and myocardial infarction, ischemic stroke, arrhythmia, all cardiovascular or cerebrovascular accident events, and atherosclerosis. 3. Medication: centrally acting anti-adrenergic agents, ą-blockers, thiazide-type diuretics, ß-blockers, calcium channel blockers, angiotensin-converting enzyme inhibitors, angiotensin II receptor antagonists, anti-diabetic therapies (metformin, sulfonylureas, acarbose, TZD, DPP4, GLP1, SGLT2, meglitinides, and insulin).

A total of 95,627 patients diagnosed with diabetes between 2001 and 2018 fulfilled the study criteria (Fig. 1). The mean age was 62.04 ± 12.65 years, 48% of the included patients were female, 1.6% had cholangitis, 67.4% took metformin, and the mean value of glycated hemoglobin was 8.42 ± 2.25%. Table 1 shows their clinical characteristics before and after PSM.

**Table 1:** Baseline characteristics before and after propensity score matching.

	Unmatched cohort (n = 95,627)				PSM cohort (n = 63,500)			
	Total (n = 95,627) n (%)	Aspirin users (n = 43,247) n (%)	Non-users (n = 52,380) n (%)	SMD*	Total (n = 63,500) n (%)	Aspirin users (n = 31,750) n (%)	Non-users (n = 31,750) n (%)	SMD*
**Sex**								
Male	49,118 (51.36)	22,505 (52.04)	26,613 (50.81)	0.02	32,157 (50.64)	16,151 (50.87)	16,006 (50.41)	0.01
Female	46,509 (48.64)	20,742 (47.96)	25,767 (49.19)	0.02	31,343 (49.36)	15,599 (49.13)	15,744 (49.59)	0.01
**Age at diabetes diagnosis, years**				0.16				0.03
20–64	54,508 (57.00)	22,796 (52.71)	31,712 (60.54)		36,201 (57.01)	17836 (56.18)	18,365 (57.84)	
≥ 65	41,119 (43.00)	20,451 (47.29)	20,668 (39.46)		27,299 (42.99)	13,914 (43.82)	13,385 (42.16)	
**Baseline comorbid condition**								
Liver cirrhosis	538 (0.56)	78 (0.18)	460 (0.88)	0.10	162 (0.26)	75 (0.24)	87 (0.27)	0.01
Unspecified liver injury	28 (0.03)	6 (0.01)	22 (0.04)	0.02	12 (0.02)	6 (0.02)	6 (0.02)	0.00
Hepatobiliary malignancy	328 (0.34)	46 (0.11)	282 (0.54)	0.08	75 (0.12)	44 (0.14)	31 (0.10)	0.01
Gastric cancer	419 (0.44)	92 (0.21)	327 (0.62)	0.06	166 (0.26)	86 (0.27)	80 (0.25)	0.00
Colon cancer	1222 (1.28)	370 (0.86)	852 (1.63)	0.07	641 (1.01)	319 (1.00)	322 (1.01)	0.00
Hepatocellular carcinoma	2588 (2.71)	381 (0.88)	2207 (4.21)	0.21	738 (1.16)	374 (1.18)	364 (1.15)	0.00
Metastatic cancers	3132 (3.28)	364 (0.84)	2768 (5.28)	0.26	647 (1.02)	361 (1.14)	286 (0.90)	0.02
Other malignancies	9039 (9.45)	2232 (5.16)	6807 (13.00)	0.28	3972 (6.26)	2065 (6.50)	1907 (6.01)	0.02
Cholangitis	1011 (1.06)	275 (0.64)	736 (1.41)	0.08	478 (0.75)	245 (0.77)	233 (0.73)	0.00
Cholelithiasis	3223 (3.37)	1180 (2.73)	2043 (3.90)	0.07	1906 (3.00)	939 (2.96)	967 (3.05)	0.01
Hepatitis B	2562 (2.68)	505 (1.17)	2057 (3.93)	0.18	933 (1.47)	487 (1.53)	446 (1.40)	0.01
Hepatitis C	2623 (2.74)	551 (1.27)	2072 (3.96)	0.17	1037 (1.63)	522 (1.64)	515 (1.62)	0.00
Other viral hepatitis	97 (0.10)	27 (0.06)	70 (0.13)	0.02	46 (0.07)	22 (0.07)	24 (0.08)	0.00
Chronic kidney disease	7530 (7.87)	3509 (8.11)	4021 (7.68)	0.02	4957 (7.81)	2609 (8.22)	2348 (7.40)	0.03
Cardiovascular events								
Ischemic heart disease and myocardial infarction	13,632 (14.26)	9345 (21.61)	4287 (8.18)	0.38	7548 (11.89)	3852 (12.13)	3696 (11.64)	0.02
Ischemic stroke	8400 (8.78)	5896 (13.63)	2504 (4.78)	0.31	4671 (7.36)	2433 (7.66)	2238 (7.05)	0.02
Arrythmia	5372 (5.62)	2951 (6.82)	2421 (4.62)	0.09	3626 (5.71)	1868 (5.88)	1758 (5.54)	0.01
Any cardiovascular or cerebrovascular accident event	28,975 (30.30)	18,226 (42.14)	10,749 (20.52)	0.48	17,760 (27.97)	9142 (28.79)	8618 (27.14)	0.04
Atherosclerosis	1861 (1.95)	1133 (2.62)	728 (1.39)	0.09	1152 (1.81)	621 (1.96)	531 (1.67)	0.02
**Prior medications**								
Anti-diabetic therapy								
Metformin	64,474 (67.42)	29,688 (68.65)	34,786 (66.41)	0.05	43,382 (68.32)	21,612 (68.07)	21,770 (68.57)	0.01
Sulfonylurea	49,442 (51.70)	24,566 (56.80)	24,876 (47.49)	0.19	33,779 (53.20)	16,779 (52.85)	17,000 (53.54)	0.01
Acarbose	6637 (6.94)	3205 (7.41)	3432 (6.55)	0.03	4489 (7.07)	2266 (7.14)	2223 (7.00)	0.01
Thiazolidinedione	4730 (4.95)	2848 (6.59)	1882 (3.59)	0.14	3164 (4.98)	1568 (4.94)	1596 (5.03)	0.00
DPP4 inhibitor	4543 (4.75)	1637 (3.79)	2906 (5.55)	0.08	2643 (4.16)	1398 (4.40)	1245 (3.92)	0.02
GLP-1 agonist	11 (0.01)	4 (0.01)	7 (0.01)	0.00	7 (0.01)	4 (0.01)	3 (0.01)	0.00
SGLT2 inhibitor	135 (0.14)	43 (0.10)	92 (0.18)	0.02	60 (0.09)	39 (0.12)	21 (0.07)	0.02
Meglitinides	7584 (7.93)	3718 (8.60)	3866 (7.38)	0.04	5177 (8.15)	2620 (8.25)	2557 (8.05)	0.01
Insulins	2174 (2.27)	428 (0.99)	1746 (3.33)	0.16	835 (1.31)	423 (1.33)	412 (1.30)	0.00
Centrally acting anti-adrenergic agents	332 (0.35)	162 (0.37)	170 (0.32)	0.01	216 (0.34)	109 (0.34)	107 (0.34)	0.00
α-blockers	1255 (1.31)	562 (1.30)	693 (1.32)	0.00	793 (1.25)	414 (1.30)	379 (1.19)	0.01
Thiazide-type diuretics	11,343 (11.86)	5609 (12.97)	5734 (10.95)	0.06	7364 (11.60)	3674 (11.57)	3690 (11.62)	0.00
ß-blockers	18,784 (19.64)	10,258 (23.72)	8526 (16.28)	0.19	12,035 (18.95)	6037 (19.01)	5998 (18.89)	0.00
Calcium channel blockers	27,997 (29.28)	14,595 (33.75)	13,402 (25.59)	0.18	18,935 (29.82)	9595 (30.22)	9340 (29.42)	0.02
Angiotensin-converting enzyme inhibitors	10,628 (11.11)	6523 (15.08)	4105 (7.84)	0.23	6915 (10.89)	3386 (10.66)	3529 (11.11)	0.01
Angiotensin II receptor antagonists	20,967 (21.93)	11,678 (27.00)	9289 (17.73)	0.22	14,790 (23.29)	7393 (23.29)	7397 (23.30)	0.00
**HbA1c value**	8.42 ± 2.25	8.40 ± 2.17	8.43 ± 2.33	0.26	8.45 ± 2.25	8.46 ± 2.21	8.45 ± 2.28	0.03

DPP4, dipeptidyl peptidase 4 inhibitors; GLP1, glucagon-like peptide-1 analogues; HbA1c, glycated hemoglobin; SGLT2, sodium-glucose co-transporter 2 inhibitors; SMD, standardized mean differences.

*an SMD < 0.1 was considered well balance.

In the PSM cohort, the baseline comorbid conditions and prior medications were not significantly different between the aspirin users and non-users. The mean follow-up time was 5.69 years (Supplementary Table S1-1). Among aspirin users, 14.2% and 35.8% were exposed to aspirin for 1–90 days and > 90 days during the follow-up, respectively (Supplementary Table S1-2). Table 2 presents the concomitant medications used during the study follow-up. Among the aspirin-users, 22.0% had taken clopidogrel for > 90 days, 57.8% H2-blockers or proton pump inhibitors for > 5 days, and 76.7% metformin for > 90 days during the follow-up. Th ere was a small number of patients with unspecified liver injury (n = 28), other viral hepatitis (n = 97), or taking centrally acting anti-adrenergic agents (n = 332) and they were not considered in further analyses.

**Table 2:** Concomitant medications used during follow-up in the matched cohort.

	Aspirin users (n = 31,750) n (%)	Non-users (n = 31,750) n (%)	*p* value
**Clopidogrel**			< 0.01
Non-exposure	21,308 (67.11)	29,350 (92.44)	
1–90 days	3447 (10.86)	657 (2.07)	
> 90 days	6995 (22.03)	1743 (5.49)	
**Ticagrelor**			< 0.01
Non-exposure	30,322 (95.50)	31,726 (99.92)	
Yes	1428 (4.50)	24 (0.08)	
**Other anti-platelet agents**			<0.01
Non-exposure	28,641 (90.21)	30,904 (97.34)	
Yes	3109 (9.79)	846 (2.66)	
**H2-blocker or proton pump inhibitors **			< 0.01
Non-exposure	11,951 (37.64)	15,304 (48.20)	
1–4 days	1445 (4.55)	1380 (4.35)	
5–30 days	3109 (9.79)	3334 (10.50)	
31–90 days	3652 (11.50)	3425 (10.79)	
> 90 days	11,593 (36.51)	8307 (26.16)	
**Metformin**			< 0.01
Non-exposure	4506 (14.19)	5139 (16.19)	
1–30 days	1353 (4.26)	2090 (6.58)	
31–90 days	1527 (4.81)	2243 (7.06)	
> 90 days	24,364 (76.74)	22,278 (70.17)	

### KP liver abscess and in-hospital mortality

**Figure 2:**
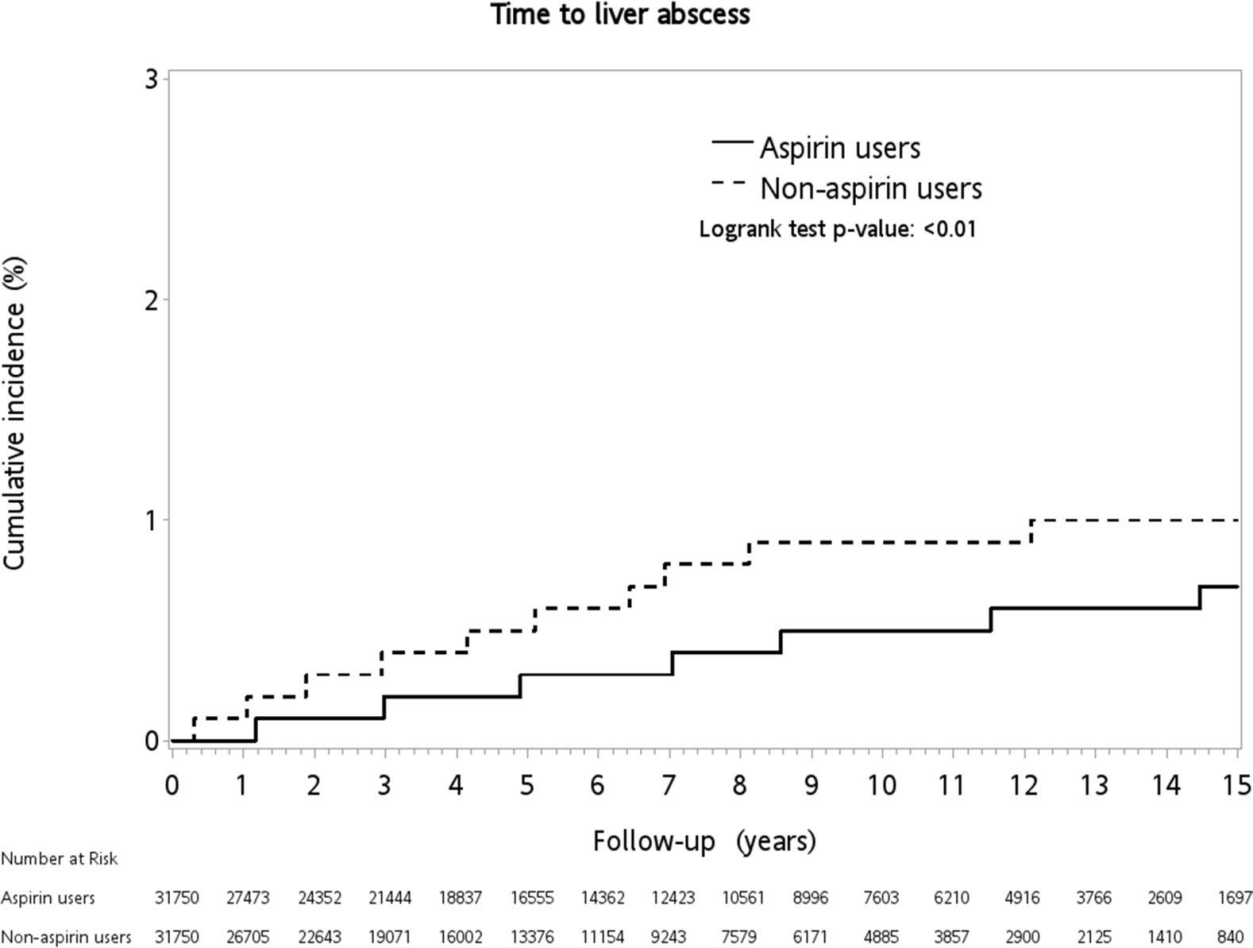
Kaplan–Meier curve of the incidence of *Klebsiella pneumoniae* liver abscess in diabetic patients with or without aspirin use.

The cumulative incidence of KP-PLA was 0.35% in the primary cohort and 0.41% in the PSM cohort over the 15 years of follow-up. In the primary cohort, the incidence of KP-PLA was significantly higher in the non-user group compared with the aspirin user group (0.57% *vs.* 0.29%, *p* < 0.01) (Table 3). This significant difference in the incidence of KP-PLA remained between the two groups after matching (0.50% in the non-users *vs.* 0.31% in the aspirin users; log-rank test, *p* < 0.01) (Fig. 2).

**Table 3:** Comparisons of incidences of *K. pneumoniae* liver abscess, invasive *K. pneumoniae* liver abscess syndrome and mortality between aspirin users and non-users in unmatched cohort and propensity-score matched (PSM) cohort.

	Unmatched cohort (n = 95,627)			PSM cohort (n = 63,500)		
	Aspirin users (n = 43,247) n (%)	Non-users (n = 52,380) n (%)	*p* value	Aspirin users (n = 31,750) n (%)	Non-users (n=31,750) n (%)	*p* value
***K. pneumoniae *liver abscess**	124 (0.29)	298 (0.57)	< 0.01	98 (0.31)	159 (0.50)	< 0.01
**Invasive K. pneumoniae liver abscess syndrome**	5 (0.01)	13 (0.02)	0.14	5 (0.02)	9 (0.03)	0.29
Extrahepatic complications						
Endophthalmitis	5 (0.01)	2 (0.00)		5 (0.02)	0 (0.00)	
Brain abscess	0 (0.00)	1 (0.00)		0 (0.00)	1 (0.00)	
Bacterial meningitis	0 (0.00)	4 (0.01)		0 (0.00)	3 (0.01)	
Lung abscess	0 (0.00)	5 (0.01)		0 (0.00)	4 (0.01)	
Abscess of the prostate	0 (0.00)	1 (0.00)		0 (0.00)	1 (0.00)	
**In-hospital mortality**	3976 (9.19)	4932 (9.42)	0.24	2804 (8.83)	2426 (7.64)	< 0.01

**Figure 3:**
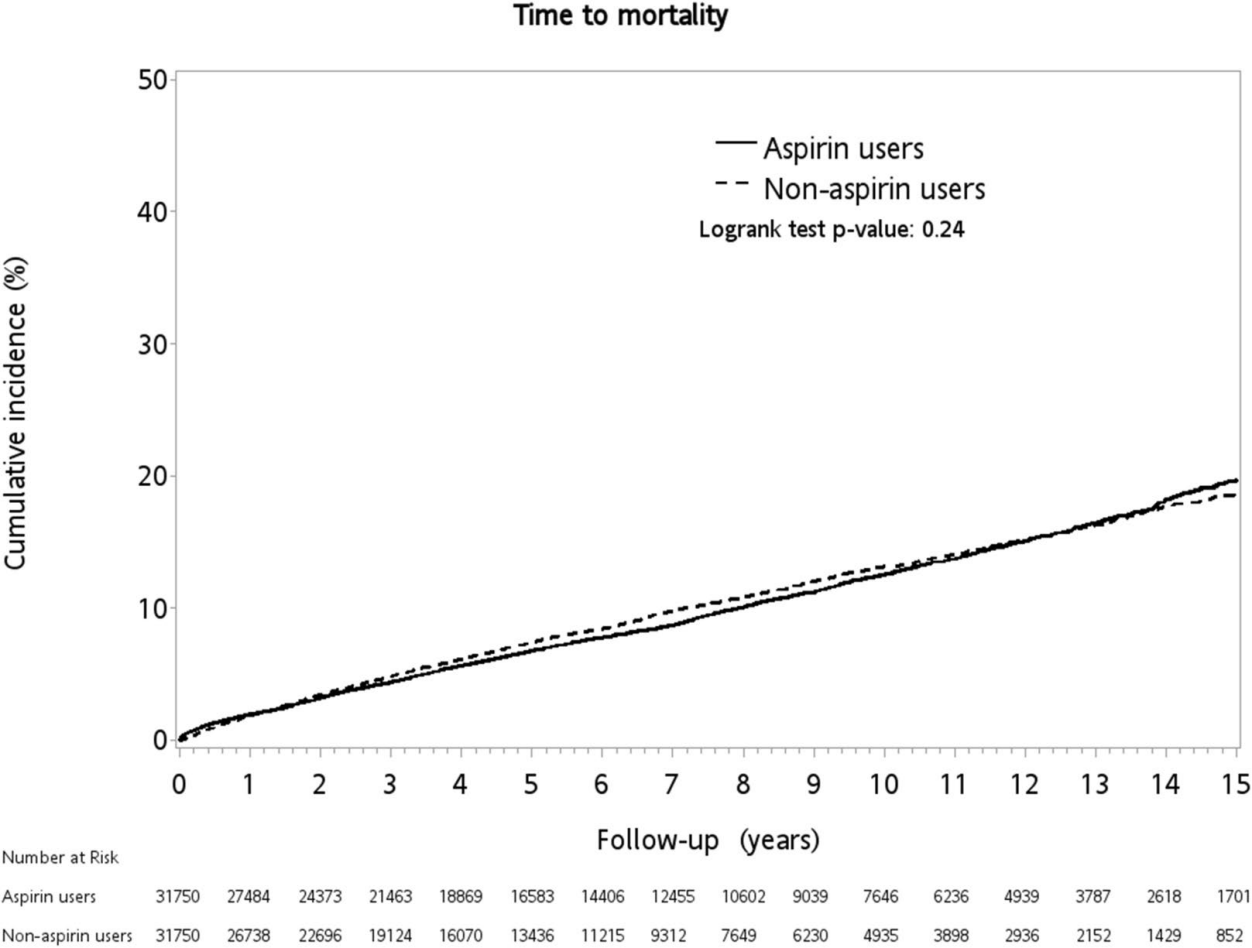
Kaplan–Meier curve of in-hospital mortality in diabetic patients with or without aspirin use.

The incidence of invasive KP-PLA syndrome was low (n = 18 in the primary cohort and n = 14 in the PSM cohort) and was not significantly different between the aspirin user and non-user groups in both primary and PSM cohorts (Table 3). In-hospital mortality was 9.3% in the primary cohort and 8.2% in the PSM cohort over the study period. Although the all-cause mortality rate was slightly higher in the aspirin user group compared with the non-user group (8.83% *vs.*7.64%, *p* < 0.01) in the PSM cohort, it was not significantly different over the study period (log-rank test, *p* = 0.24) (Fig. 3).

Duration of aspirin exposure was significantly associated with the risk of KP-PLA, after controlling for baseline characteristics and concomitant medications during the follow-up (Table 4). The risk of KP-PLA was significantly lower among patients with > 90 days of aspirin exposure (aHR, 0.67; 95%CI, 0.50–0.90; *p* = 0.01) compared with non-users; however, there was no significant difference between those with < 90 days of aspirin exposure (aHR, 0.87; 95%CI, 0.56–1.34; *p* = 0.52) and non-users.

**Table 4:** Risk of *K. pneumoniae* liver abscess adjusted for in-hospital mortality as a competing risk.

	Adjusted HR	95%CI	*p* value
**Aspirin**			
Non-exposure	Ref		
1–90 days	0.87	(0.56–1.34)	0.52
> 90 days	0.67	(0.50–0.90)	0.01
**Sex**			
Male	Ref		
Female	0.58	(0.45–0.75)	< 0.01
**Age group**			
20–64 years	Ref		
≥ 65 years	0.89	(0.67–1.17)	0.39
**Comorbidities**			
Liver cirrhosis	2.44	(0.31–18.93)	0.39
Hepatobiliary malignancy	2.88	(0.38–21.80)	0.31
Gastric cancer	2.24	(0.31–16.19)	0.42
Colon cancer	0.47	(0.07–3.41)	0.46
Hepatocellular carcinoma	2.08	(0.73–5.96)	0.17
Metastatic cancers	0.81	(0.11–6.14)	0.84
Other malignancies	0.88	(0.47–1.63)	0.68
Cholangitis	3.00	(1.21–7.41)	0.02
Cholelithiasis	1.64	(0.87–3.09)	0.12
Hepatitis B	0.81	(0.25–2.67)	0.73
Hepatitis C	0.64	(0.15–2.66)	0.54
Chronic kidney disease	0.74	(0.40–1.37)	0.34
Any cardiovascular or cerebrovascular accident event	0.93	(0.68–1.28)	0.67
Atherosclerosis	0.63	(0.16–2.53)	0.51
**Prior medications**			
α-blockers	0.40	(0.06–2.86)	0.36
Thiazide-type diuretics	1.00	(0.65–1.53)	0.99
ß-blockers	1.01	(0.71–1.45)	0.95
Calcium channel blockers	0.92	(0.67–1.26)	0.60
Angiotensin-converting enzyme inhibitors	0.88	(0.58–1.32)	0.54
Angiotensin II receptor antagonists	1.00	(0.73–1.37)	0.99
**HbA1c value**			
Lack of HbA1c data	0.15	(0.02–1.09)	0.06
HbA1c < 7.0%	Ref		
7.0% ≤ HbA1c < 8.5%	1.18	(0.82–1.71)	0.38
HbA1c ≥ 8.5%	1.80	(1.30–2.50)	< 0.01
**Concomitant medications**			
Clopidogrel			
Non-exposure	Ref		
1–90 days	1.06	(0.59–1.89)	0.84
> 90 days	0.41	(0.22–0.75)	< 0.01
Ticagrelor			
Non-exposure	Ref		
Yes	1.44	(0.45–4.62)	0.54
Other anti-platelet agents			
Non-exposure	Ref		
Yes	0.98	(0.57–1.71)	0.96
H_2_-blocker or proton pump inhibitors			
Non-exposure	Ref		
1–4 days	0.73	(0.41–1.29)	0.28
5–30 days	0.51	(0.33–0.79)	< 0.01
31–90 days	0.27	(0.15–0.48)	< 0.01
> 90 days	0.30	(0.21–0.43)	< 0.01
Metformin			
Non-exposure	Ref		
1–30 days	0.96	(0.55–1.67)	0.89
31–90 days	0.62	(0.34–1.14)	0.12
> 90 days	0.50	(0.35–0.72)	< 0.01

CI, confidence interval; HbA1c, glycated hemoglobin; HR, hazard ratio.

*Due to the small number of patients, unspecified liver injury, other viral hepatitis (exclude hepatitis B and hepatitis C), and centrally acting anti-adrenergic agents were not considered in the analyses.

Compared with male patients, females were associated with a significantly lower risk of KP-PLA (aHR, 0.58; 95%Cl, 0.45–0.75; *p* < 0.01) (Table 4). Diabetic patients with cholangitis (aHR, 3.00; 95%Cl, 1.21–7.41; *p* = 0.02), and those who had a poorly controlled glucose status with a glycated hemoglobin value ≥ 8.5% (aHR, 1.80; 95%Cl, 1.30–2.50; *p* < 0.01) were associated with a significantly greater risk of developing KP-PLA. In contrast, taking clopidogrel for > 90 days (aHR, 0.41; 95%Cl, 0.22–0.75; *p* < 0.01), taking metformin for > 90 days (aHR, 0.50; 95%Cl, 0.35–0.72; *p* < 0.01), or taking H_2_-blockers or proton pump inhibitors for > 5 days were associated with a significantly reduced risk of developing KP-PLA.

## Discussion

The current study demonstrates that use of aspirin for > 90 days was associated with a lower risk of KP-PLA in diabetic patients without an increase in mortality. Although the risk of KP-PLA decreased with aspirin use, that of invasive KP-PLA syndrome did not. This was probably due to the low prevalence of metastatic infection in both aspirin users and non-users. Aspirin has been reported to inhibit the production of KP CPS, which is important to its virulence because of its anti-phagocytic effect against macrophages and neutrophils^12,21^. Platelet-monocyte aggregation is also involved in the pathogenesis of KP-PLA in diabetic patients, which was shown to be decreased by aspirin^22^.

In addition to aspirin use, our study revealed that taking clopidogrel reduced the risk of KP-PLA. Chao *et* *al.* found that taking aspirin and clopidogrel for 1–12 months could change the intestinal flora; the number of *Lactobacillales* and *Lactobacillaceae* within the flora increased at the order level and family level, respectively^23^. The development of KP-PLA is associated with changes in the gut microbiota, especially a reduction in the *Lactobacillales* and *Lactobacillaceae* flora^24^. Some species of *Lactobacillus* have been proved to inhibit KP in vitro^25^. On the other hand, ticagrelor and other anti-platelet agents, such as dipyridamole, tirofiban, or cilostazol, were not demonstrated to change the gut microbiota. Our study also did not find that these agents were associated with a reduced risk of KP-PLA.

Our study demonstrates that using H_2_receptor antagonists or proton pump inhibitors for a minimum of 5 days was associated with a lower risk of KP-PLA. Previous studies had shown that treatment with proton pump inhibitors increased *Lactobacillales* not only in animal models but also in healthy individuals^26,27^. There was no direct evidence for changes in the gut microbiota after using H_2_receptor antagonists, yet it seemed that the microflora was still affected by those medications^28,29^. In contrast to our findings, Wang et al*.* used the National Health Insurance Research Database (NHIRD) in Taiwan and discovered that using proton pump inhibitors for > 5 cumulative defined daily doses increased the risk of PLA^30^. Our study, using the CGRD, could accurately identify diabetic patients with KP-PLA, and this information cannot be gathered from the NHIRD. After propensity-score matching of the glycated hemoglobin value, we found that using metformin for > 90 days could considerably reduce the risk of KP-PLA. Individuals taking metformin for 2 months were shown to have changes in their gut microbiota, especially in the growth of *Bifidobacterium*, of which some strains seemed to have antibacterial activity against enteropathogenic bacteria^31,32^. Other studies revealed increased amount of *Lactobacillus* with metformin treatment^33,34^. These results indicate that metformin could alter the human gut microbiota; however, how it affects KP colonization in the human gut warrants more investigations.

Compared to male patients, female patients had a lower incidence of PLA, not only in our study but also in previous studies using the Taiwan NHIRD^6,18^. Biliary tract disease was reported to be associated with PLA and was much more likely to be associated with polymicrobial infections, including *Escherichia coli, Enterococcus* and *Klebsiella* species^6,35^. Our study supported the finding that diabetic patients who previously had cholangitis were at greater risk of KP-PLA. Patients with a glycated hemoglobin level > 7% were associated with impaired phagocytosis of KP serotypes K1 and K2 and a higher incidence of PLA^17,36^*.* Our findings support the theory that poor glycemic control, defined as a glycated hemoglobin level more than 8.5%, was associated with a significantly increased risk of KP-PLA.

A strength of the current study was that it was performed using the CGRD, a database gathered from two medical centers and five local hospitals, with high overall coverage of individuals seeking care reimbursed by the National Health Insurance in Taiwan^37^. It could provide detailed information on the patients’ diagnosis, medications, laboratory and examination data. However, there were still some limitations. Previous studies demonstrated that patients in the CGRD had more severe comorbidities than the general population^37^. Therefore, we used propensity-score matching to minimize the differences in comorbidities. In addition, patients might return to their primary care facilities, not the Chang Gung Memorial Hospitals, for their long-term follow‐up after their disease stabilized. This might cause bias in the event rate and mortality. To eliminate the bias of loss to follow‐up, we excluded patients who didn’t have any medical records for one year prior to their diagnosis of diabetes. The incidence of invasive KP-PLA syndrome was previously reported to be 15% in Taiwan^12^; however, it was about 4.2% (18/422) and 5.4% (14/257) in unmatched cohort and PSM cohort, respectively in our study. The prevalence of invasive KP-PLA syndrome might be under-estimated in our study because it was not a routine to screen for any metastatic infection in each patient with KP-PLA in clinical practice. Finally, it was a retrospective study and there might be some confounding factors that were not measured in the analysis despite adjustment by propensity-score matching.

In conclusion, we found that females, taking clopidogrel or metformin for ≥ 90 days, and taking H2-blockers or proton pump inhibitors for ≥ 5 days, and taking aspirin > 90 days were associated a reduced risk of KP-PLA in diabetic patients without increasing mortality. In contrast, aspirin had no effect on invasive KP-PLA syndrome in these patients. Further randomized controlled trial might be required to establish the role of aspirin in prevention of KP-PLA in diabetic patients.

## Methods

### Data source

The present retrospective cohort study was performed using the Chang Gung Research Database (CGRD), which is an electronic health records dataset from a large healthcare delivery system at Chang Gung Memorial Hospital in Taiwan, which includes two medical centers and five local hospitals^38^. The CGRD covers approximately 10% of all health services within the Taiwan National Health Insurance program, which is a single-payer nationwide health insurance program that covers over 99% of Taiwan’s population^39^. The CGRD contains detailed diagnostic, prescription, and laboratory test result information from the emergency department, inpatient, and outpatient settings. The current study was approved by the Institutional Review Board of Chang Gung Medical Foundation, Taipei, Taiwan (201900920B0). All personal identifiable information was anonymized, and, therefore, the need for informed consent was waived. All methods were carried out in accordance with relevant guidelines and regulations.

### Study cohort

We identified patients from the CGRD who had DM and were being newly treated with anti-diabetic therapy (ADT) (*i.e.*, insulin, GLP-1 agonists or oral hypoglycemic agents) between January 1st 2001 and December 31st 2018. The information of the diabetic patients prescribed ADT was retrieved from the CGRD via their Anatomical Therapeutic Chemical (ATC) codes in the outpatient setting (Supplementary Table S2). The date the patients were first prescribed ADT was defined as the date they received the diagnosis of DM. Patients were excluded if they were aged < 20 years at the date of their diagnosis of DM; or were diagnosed with amebic liver abscess and PLA in the same hospitalization; had a prior hospitalization for PLA; had a history of aspirin use; or had no hospital admission or visit within a one-year prior to their diagnosis (Fig. 1). In addition, for outcome measurements, only diabetic patients who had at least one hospitalization during the follow-up were included in the study cohort.

Included patients were classified into aspirin users and non-users. Aspirin users were defined as those who started taking aspirin following the date of their diabetes diagnosis, and the date of their first aspirin prescription was defined as the study index date. Propensity-score matching (PSM) with a 1:1 ratio was employed to balance the distribution of baseline characteristics between aspirin users and non-users who were alive at the time aspirin was first prescribed to their matched users. The details of the PSM approach are described in the following statistical analysis section.

### Outcomes

The primary outcome of interest was KP-PLA, as defined by an International Classification of Disease, 9th and 10th Clinical Modification (ICD-9/10-CM) code for liver abscess at the time of hospital discharge (Supplementary Table S3). KP infection was defined as having a blood, pus or abscess culture that yielded the growth of KP during the hospital stay. The secondary outcome was invasive KP-PLA syndrome, which was a subgroup of KP-PLA, defined as KP-PLA with a metastatic infection, such as endophthalmitis, brain abscess, intra-spinal abscess, extradural and subdural abscess, bacterial meningitis, lung abscess, osteomyelitis, or an abscess of the prostate (Supplementary Table S3)^12^. Any cause of in-hospital mortality was assessed in the analysis. Every patient was followed from the study index date until an event of interest occurred, loss to follow-up, or the last date of the dataset (December 31st, 2018), whichever occurred first.

### Covariates

Baseline infection risk, age, sex, co-morbidities including liver cirrhosis, unspecified liver injury, hepatobiliary malignancy, gastric cancer, colon cancer, hepatocellular carcinoma, metastatic cancers, other malignancies, cholangitis, cholelithiasis, hepatitis B, hepatitis C, other viral hepatitis, chronic kidney disease, ischemic heart disease and myocardial infarction, ischemic stroke, arrythmia, any cardiovascular or cerebrovascular disease, or atherosclerosis were identified using the ICD-9/10-CM codes from outpatient visits or hospital discharges within 1 year prior to the study index date (Supplementary Table S3).

Prior medical use of centrally acting anti-adrenergic agents, alpha- and beta-blockers, thiazide-type diuretics, calcium channel blockers, angiotensin-converting enzyme inhibitors, and angiotensin II receptor antagonists within 1 year prior to the index date was identified by ATC codes (Supplementary Table S2). In addition, anti-platelet agents (clopidogrel, ticagrelor and others), H2 receptor antagonists (H2-blockers), or proton pump inhibitors prescribed during the study follow-up were considered as concomitant medications (Supplementary Table S2).

### Statistical analysis

Continuous variables were reported as the mean and standard deviation (SD) and categorical variables were reported as the number and percentage. The individual’s propensity-score was calculated using multivariate logistic regression, including class of ADT (n = 9), baseline comorbid conditions, prior medications, glycated hemoglobin, and interval between diabetes diagnosis and first aspirin prescription (39 variables in total). To minimize the possible effect of immortal time bias, the length of time from diabetes diagnosis to the index date (aspirin initiation) was ascertained and randomly selected non-users who were followed during the same period. The balance between aspirin user and non-user groups was assessed using the absolute standardized mean differences of individual covariates at baseline before and after matching, using a threshold of < 0.1 to indicate balance.

The cumulative incidence of an event of interest between the two groups was analyzed using the Kaplan–Meier approach with a log-rank test. Death-censored Cox proportional hazards regression was used to estimate the adjusted hazard ratio (aHR) with 95% confidence intervals (CIs) for KP-PLA or invasive KP-PLA syndrome in the PSM cohort. The level of significance was set as 5% (*p* < 0.05) for two-sided tests. Statistical analyses and data management were performed using SAS software version 9.4 (SAS Institute Inc. Cary, NC, USA).

## Supplementary information

Supplementary information.

## Data Availability

The datasets generated during and/or analyzed during the current study are available from the corresponding author on reasonable request.
