# Supplementary material for: The impact of aspirin on Klebsiella pneumoniae liver abscess in diabetic patients
Source: Sci Rep. 2020 Dec 7;10:21329. doi: 10.1038/s41598-020-78442-8 (PMC7721809; doi:10.1038/s41598-020-78442-8)
Supplement: Supplementary file 1 — Supplementary information. [file 41598_2020_78442_MOESM1_ESM.docx]

**The impact of aspirin on *Klebsiella pneumoniae* liver abscess in diabetic patients**

Chien-Hsiang Tai^a^, Chien-Ning Hsu^b,c^, Shih-Cheng Yang^d^, Cheng-Kun Wu^d,e^ Chih-Ming Liang^d,e^, Wei-Chen Tai^d,e^, Seng-Kee Chuah^d,e^ and Chen-Hsiang Lee^a,e,^*

^a^Division of Infectious Diseases, Department of Internal Medicine, Kaohsiung Chang Gung Memorial Hospital, Kaohsiung, Taiwan.

^b^Department of Pharmacy, Kaohsiung Chang Gung Memorial Hospital, Kaohsiung, Taiwan.

^c^School of Pharmacy, Kaohsiung Medical University, Kaohsiung, Taiwan.

^d^Division of Hepato-Gastroenterology, Department of Internal Medicine, Kaohsiung Chang Gung Memorial Hospital, Kaohsiung, Taiwan.

^e^College of Medicine, Chang Gung University, Kaohsiung, Taiwan.

*Email: lee900@cgmh.org.tw (CHL)

**Supplementary Materials**

**Supplementary** **Table S1-1.** Follow-up time

**Supplementary** **Table S1-2.** Distribution of aspirin users in the PSM cohort

**Supplementary** **Table S2.** Codes and definition of medication used in the study

**Supplementary** **Table S3.** Codes for disease conditions and definitions used in the study

**Supplementary** **Table S1-1.** Follow-up time

|  | **N** | **Mean (years)** | **Standard deviation (years)** | **Minimal (years)** | **25th percentile (years)** | **Median (years)** | **75th percentile (years)** | **Maximal (years)** |
| --- | --- | --- | --- | --- | --- | --- | --- | --- |
| **Non-users** | 31750 | 5.15 | 4.19 | 0.00 | 1.70 | 4.04 | 7.76 | 15.99 |
| **Aspirin users** | 31750 | 6.22 | 4.67 | 0.00 | 2.18 | 5.30 | 9.77 | 15.99 |
| **Total** | 63500 | 5.69 | 4.47 | 0.00 | 1.91 | 4.62 | 8.75 | 15.99 |

**Supplementary** **Table S1-2.** Distribution of aspirin users in the PSM cohort

| **Aspirin** | **N** | **(%)** |
| --- | --- | --- |
| Non-exposure | 31750 | 50.0 |
| 1-90 days | 8991 | 14.2 |
| >90 days | 22759 | 35.8 |

**Supplementary** **Table S2.** Codes and definitions of medication use in the study

| **Class** |  | **ATC codes** |
| --- | --- | --- |
| Insulins and analogues |  | A10A |
| Metformin |  | A10BA02, A10BD |
| Sulfonylureas |  | A10BD02, A10BB |
| Alpha glucosidase inhibitors |  | A10BF |
| Thiazolidinediones |  | A10BG |
| Dipeptidyl peptidase 4 (DPP-4) inhibitors |  | A10BH |
| Glucagon-like peptide-1 (GLP-1) analogues |  | A10BJ |
| Sodium-glucose co-transporter 2 (SGLT2) inhibitors |  | A10BK |
| Meglitinides |  | A10BX02, A10BX03, A10BX008 |
| Centrally acting anti-adrenergic agents |  | C02A |
| α-blockers |  | C02CA |
| Thiazide-type diuretics |  | C03A, C03B, C03D, C09DX, C03EA |
| ß-blockers |  | C07, C09BX |
| Calcium channel blockers |  | C08, C09BB, C09DB |
| Angiotensin-converting enzyme inhibitors |  | C09A, C09DX |
| Angiotensin II receptor antagonists |  | C09CA |
| Clopidogrel |  | B01AC04 |
| Ticagrelor |  | B01AC24 |
| Other anti-platelet agents |  | B01AC07, B01AC09, B01AC11, B01AC17, B01AC22, B01AC23 |
| H_2_-blocker or proton pump inhibitors |  | A02BA, A02BC01, A02BC02, A02BC03, A02BC04, A02BC05, A02BC06 |

**Supplementary** **Table S3.** Codes for disease conditions and definitions in the study

| **Disease** |  | **ICD 9 code** |  | **ICD 10 code** |
| --- | --- | --- | --- | --- |
| Liver abscess |  | 572.0 |  | K75.0 |
| Endophthalmitis |  | 360.0, 360.1 |  | H44.0, H44.1 |
| Brain abscess |  | 324.0 |  | G06.0 |
| Intra-spinal abscess |  | 324.1 |  | G06.1 |
| Extradural and subdural abscess |  | 324.9 |  | G06.2 |
| Bacterial meningitis |  | 320, 322 |  | G00, G01 |
| Lung abscess |  | 513 |  | J85 |
| Osteomyelitis |  | 730 |  | M86 |
| Abscess of the prostate |  | 601.0, 601.2, 601.8, 601.9 |  | N41 |
| Liver cirrhosis |  | 789.2 + (571.2, 571.5, 571.6) |  | R16.1 + (K74.0~K74.6, K70.3, K70.2, K71.7) |
| Unspecified liver injury |  | 864.0, 864.1 |  | S36.1 |
| Hepatobiliary malignancy |  | 155.1, 156 |  | C22.1, C23, C24 |
| Gastric cancer |  | 151 |  | C16 |
| Colon cancer |  | 153 |  | C18 |
| Hepatocellular carcinoma |  | 155.0, 155.2 |  | C22.0 |
| Metastatic cancers |  | 196-198 |  | C77-C79 |
| Other malignancies |  | 140-208.91 (except 155.1, 156, 151, 153, 155.0, 155.2, 196-198) |  | C00-C96 (except C16, C18, C22.0, C22.1, C23, C24, C77-C79) |
| Cholangitis |  | 576.1 |  | K830 |
| Cholelithiasis | | 574 |  | K80 |
| Hepatitis B |  | 070.2, 070.3, V02.61 |  | B16, B18.0, B18.1, B19.1 |
| Hepatitis C |  | 070.41, 070.44, 070.51, 070.54, V02.62 |  | B17.1, B18.2, B19.2 |
| Other viral hepatitis |  | 070.0, 070.1, 070.42, 070.43, 070.49, 070.52, 070.53, 070.59, 070.6, 070.9 |  | B15, B17.0, B17.2, B17.8, B17.9,B18.8, B18.9, B19.0 |
| Chronic kidney disease |  | 585 |  | N18 |
| Ischemic heart disease and myocardial infarction |  | 410-414, 786.5 |  | I20-I25 |
| Ischemic stroke |  | 434, 435, 436 |  | I63-I68 |
| Arrythmia |  | 427 |  | I47, I48, I49 |
| Any cardiovascular and cerebrovascular accident event | | 410-414, 420-429, 430-438 |  | I20-I25, I30-I52, I60-I69 |
| Atherosclerosis | | 440, 443, 444 |  | I70, I73, I74, I75 |
